# Proof-of-Concept Evaluation of a Sock-Based Plantar Strain Sensing System for Diabetic Foot Monitoring

**DOI:** 10.3390/s26206596

**Published:** 2026-10-17

**Authors:** Rory P. Turnbull, Rachel Mason, Francesca Sairally, Jenny Corser, Giorgio Orlando, Prabhuraj D. Venkatraman, Irantzu Yoldi, Katherine Bradbury, Neil D. Reeves, Peter Culmer

**Affiliations:** 1 School of Mechanical Engineering, Faculty of Engineering and Physical Sciences, University of Leeds, Leeds LS2 9JT, UK; bs17f3s@leeds.ac.uk (F.S.); p.r.culmer@leeds.ac.uk (P.C.); 2 Medical School, Faculty of Health and Medicine, Lancaster University, Lancaster LA1 4YW, UKn.d.reeves1@lancaster.ac.uk (N.D.R.); 3 Centre for Clinical and Community Applications of Health Psychology, Shackleton Building, Highfield Campus, University of Southampton, Southampton SO17 1BJ, UK; j.c.corser@soton.ac.uk (J.C.); kjb1e08@soton.ac.uk (K.B.); 4 School of Sport and Exercise Sciences, Manchester Metropolitan University Institute of Sport, Manchester Metropolitan University, Manchester M15 6BX, UK; g.orlando@mmu.ac.uk; 5 Manchester Fashion Institute, Faculty of Arts and Humanities, Manchester Metropolitan University, Manchester M15 6BH, UK; p.venkatraman@mmu.ac.uk; 6 School of Health, Sport and Bioscience, University of East London, London E16 2RD, UK; i.yoldi@uel.ac.uk

**Keywords:** diabetic foot ulcer, shear, strain, sensors, wearables

## Abstract

Diabetic Foot Ulcers (DFUs) pose a serious health risk to people with diabetes, with a high risk of recurrence following the first DFU and a risk of foot amputation or death. It greatly impacts patient quality of life and costs the NHS up to 0.7% of its annual budget. Over the last 20 years, work has highlighted the link between DFU formation and normal forces. More recently, work has highlighted the importance of strain/shear forces, which are inherently coupled with normal forces. While limited lab-based in-shoe systems have been produced to detect shear, they alter the shoe–foot interface and are not commercially available. Additionally, consultation with people with diabetes highlighted that they may not wear shoes around the house, leaving long periods where in-shoe solutions cannot track forces. We, therefore, present the development of a novel plantar shear measuring system, the Shear Tracking for Enhanced Prevention Sock (STEPS), an instrumented garment to provide an accessible tool for people at risk of DFUs. Integrating sensors within the sock provides a solution that seamlessly integrates within the foot–shoe environment, is suitable for shoeless use, and helps mitigate adherence issues. A prototype STEPS was produced, successfully integrating printed resistive strain sensors (proxy for shear) within custom-designed socks with flexible connectivity to a welt-mounted data acquisition unit. Proof-of-concept testing characterised the system’s ability to measure plantar strain. A single-participant pilot study demonstrated that STEPS captures repeatable, gait-synchronised strain signals during walking, with a stationary noise floor below 10% of the typical per-cycle resistance response and between-repeat correlations of *R* = 0.41 and 0.78 for Sensor 1 and 2 respectively, supporting its potential to detect strain (as proxy for shear) during daily activity.

## 1. Introduction

Diabetes is a globally prevalent chronic condition, with numbers expected to reach 643 million and 783 million by 2030 and 2045, respectively [1]. Up to 25% of people with diabetes develop diabetic foot ulcers (DFUs), with infected ulcers increasing the risk of amputation [2]. A population cohort study by Walsh et al. identified a mortality rate of 5% within the first 12 months for those with new ulcers [3]. Within three years, 60% of people that have had a DFU experience recurrence [4,5]. Furthermore, DFU has a five-year mortality rate estimated at 42% [3,6]. DFUs drastically reduce patient quality of life and cost the UK approximately 0.7% of the NHS England budget, £962 million annually [7]. The social cost of DFU is even greater, estimated at up to £13.9 billion in the UK alone [8]. An estimated 450,000 people with diabetes develop a DFU in their lifetime [9]. This patient group is particularly susceptible to changes in shoe environment, with tissue structural changes observed due to diabetes [10].

DFUs are the result of foot tissue pathology and its response to external forces. Chronic hyperglycaemia in diabetes alters tissue structure [11,12,13], damages blood vessels in the extremities and reduces oxygen supply, and damages nerve endings, causing diabetic peripheral neuropathy [2,14]. These structural and physiological changes increase tissue sensitivity to forces and worsen loading, further exacerbating the condition [4,15].

**Figure 1:**
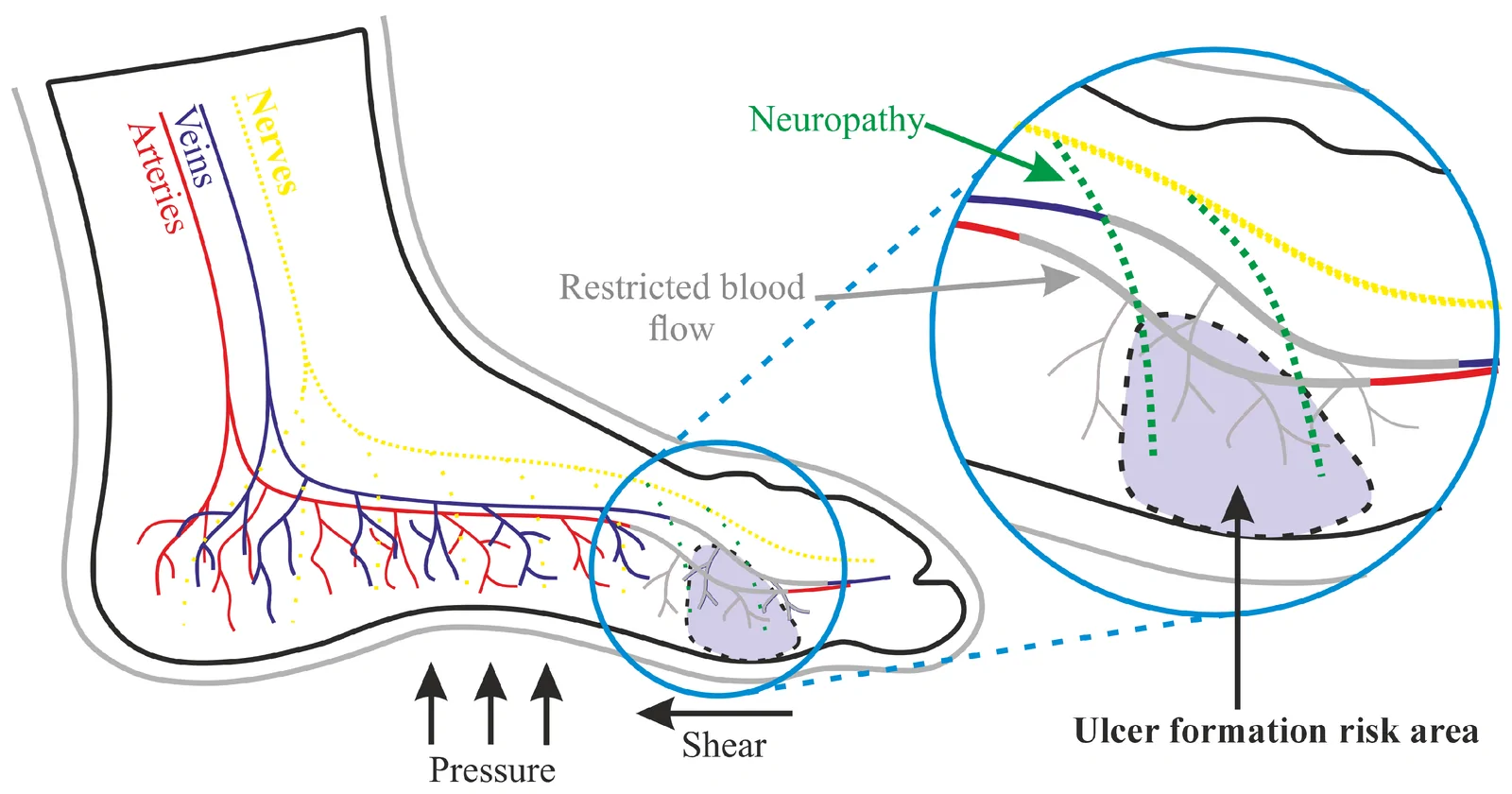
Demonstration of the conditions that lead to diabetic foot ulcer formation.

In diabetic peripheral neuropathy, oxidative stress and inflammation damage nerve endings, causing loss of sensation [16]. Typically, prolonged pressure exposure triggers subconscious postural adjustments that relieves discomfort from local tissue ischaemia and fatigue [17,18]. Without this feedback, neuropathic patients do not adjust their position, with prolonged ischaemia leading to tissue necrosis and formation of DFU (Figure 1).

Structural changes [10] and prolonged exposure to forces normal to the skin has been associated with the formation of pressure ulcers [19,20,21]. Over recent years, research has identified an important role for both normal and shear forces in the formation of DFUs [22], as highlighted in a recent systematic review and meta-analysis [23].

The current guidance on the prevention and management of DFUs is based on identifying at-risk patients, assessing the skin for at-risk regions, including checking for broken skin, managing skin moisture [24], and managing nutrition and hydration [8]. Regular offloading removes pressure and shear from vulnerable areas, supporting sufficient blood flow to the region [8,24,25,26]. In some cases, the application of supportive materials such as insoles and orthotics redistribute pressure, with extra attention paid to the bony regions [24]. Although effective methods for preventing DFU formation, they lack any form of feedback other than manually performed visual inspections. This type of guidance can be provided in clinical settings by trained professionals as standard practice, but ensuring that guidance is followed by patients once they have returned home remains a challenge. Therefore, additional tools are required to further support patients managing their condition in their daily lives. Where regular social care is impractical, wearable technologies may provide solutions.

Measurement of normal forces related to the formation of DFU is a key opportunity for DFU prevention. Currently, one of the most established methods for achieving real-time feedback is the implementation of instrumented insoles or insoles with sensors embedded within them. The technology consists of well-established sensing methods established in the 1980s [27], released in the form of products such as Novel Pedar [Munich, Bavaria, Germany] in 1986 and F-SCAN [TekSCAN, Norwood, MA, USA] in the early 1990s [27,28]. Since then, products such as the SurroSense Rx [Orpyx Medical Technologies Inc., Calgary, AB, Canada] have been released [29].

In the literature, researchers have proposed alternative pressure mapping methods such as multimodal piezo-resistive systems using a range of materials [30,31], capacitive sensors [32], and fibre optic systems [33,34]. These methods focus on the pressure perpendicular to the plantar surface. Progress has been made in the detection of shear stress in insoles with proposed methods including, but not limited to, inductive arrays [35,36], hall-effect sensors [37,38], microelectromechanical [39] and capacitive [40]. Alternative clinic-based solutions have been implemented, such as the STAMPS insole, where insole deformation provides a cumulative shear strain measure through Digital Image Correlation (DIC), helping to detect regions of the foot prone to strain [41]. Although helpful in identifying areas of high risk, real-time feedback is required for daily use.

As part of funded research, the authors coordinated patient and public involvement and engagement (PPIE) and clinical engagement sessions to better understand the requirements for fitting DFU-related measurement technology into daily life. The full findings of this research topic were published in Corser et al. [42]. A key challenge to the use of insoles is their inherent limitation to in-shoe tracking. PPIE and clinical engagement identified that patients prefer to remove their shoes for comfort in their homes, despite clinical guidance for footwear use even in the home. In the case of insoles, this interrupts foot health monitoring. In addition, insoles limit the selection of shoes available to patients due to the required depth and, in many cases, will not be compatible with prescribed orthotics. These two factors directly impact patient therapeutic adherence. Recognising that patients are likely to remove shoes inside, clinicians recommend patients wear socks to protect their feet [42].

In recent years, sensor technology has been developed, and instrumented socks, or “smart socks”, have been commercially released. DANU Sports [Dublin, Ireland] and Sensoria [Sensoria Fitness, Redmond, WA, USA] monitor vertical plantar pressure, focusing primarily on athletic performance. Similar approaches have been implemented in research settings, using various piezo-resistive materials bonded to the plantar surface of the sock [43,44,45,46]; these focused on gait monitoring. The Palarum [Lebanon, OH, USA] uses e-textiles in its fall prevention system, which reports contact with the floor. Other technologies implemented in socks include early warning systems for DFU formation using temperature measurement [47], such as Siren socks [San Francisco, CA, USA] and Milbotix [Chipping Norton, Oxfordshire, UK]. Many factors and physical conditions give rise to DFU formation, including temperature and pressure. To the best of our knowledge, no sock-based system has been designed and developed to detect shear or strain for research or commercial use. Given the importance of shear strain in DFU formation, our research has focused on developing a system capable of measuring plantar shear.

This paper proposes the Shear Tracking for Enhanced Prevention Sock (STEPS). A wearable system to assess DFU risk factors and thus prevent their formation, where the strain is taken as a proxy of the shear on the skin. In Section 2, we describe the development of the design of the wearable system. In Section 3, we evaluate system performance during a proof-of-concept case study. In Section 4, we reflect on system performance and explore areas for further research before highlighting the benefits of our approach compared to systems currently available in Section 5.

## 2. Materials and Methods

This section sets out the design and system development process used in the production of the STEPS wearable system.

### 2.1. Design Process

The SOCKSESS project set out to produce a wearable device to prevent DFU formation. The team used a human-centred approach which embraced design thinking and co-design [48]. In parallel to exploratory system conceptual development, the authors participated in a series of PPIE workshops, the key findings of which are: there is a clear appetite for this technology among patient groups; the importance of accessible information related to the formation of DFU and how to act in response to symptoms; the adoption of STEPS would depend on the perceived “burden” of use; and details on what makes a sock comfortable to wear for someone with diabetes (See Section 2.3). The detailed report by Corser et al. is available at [42].

**Figure 2:**
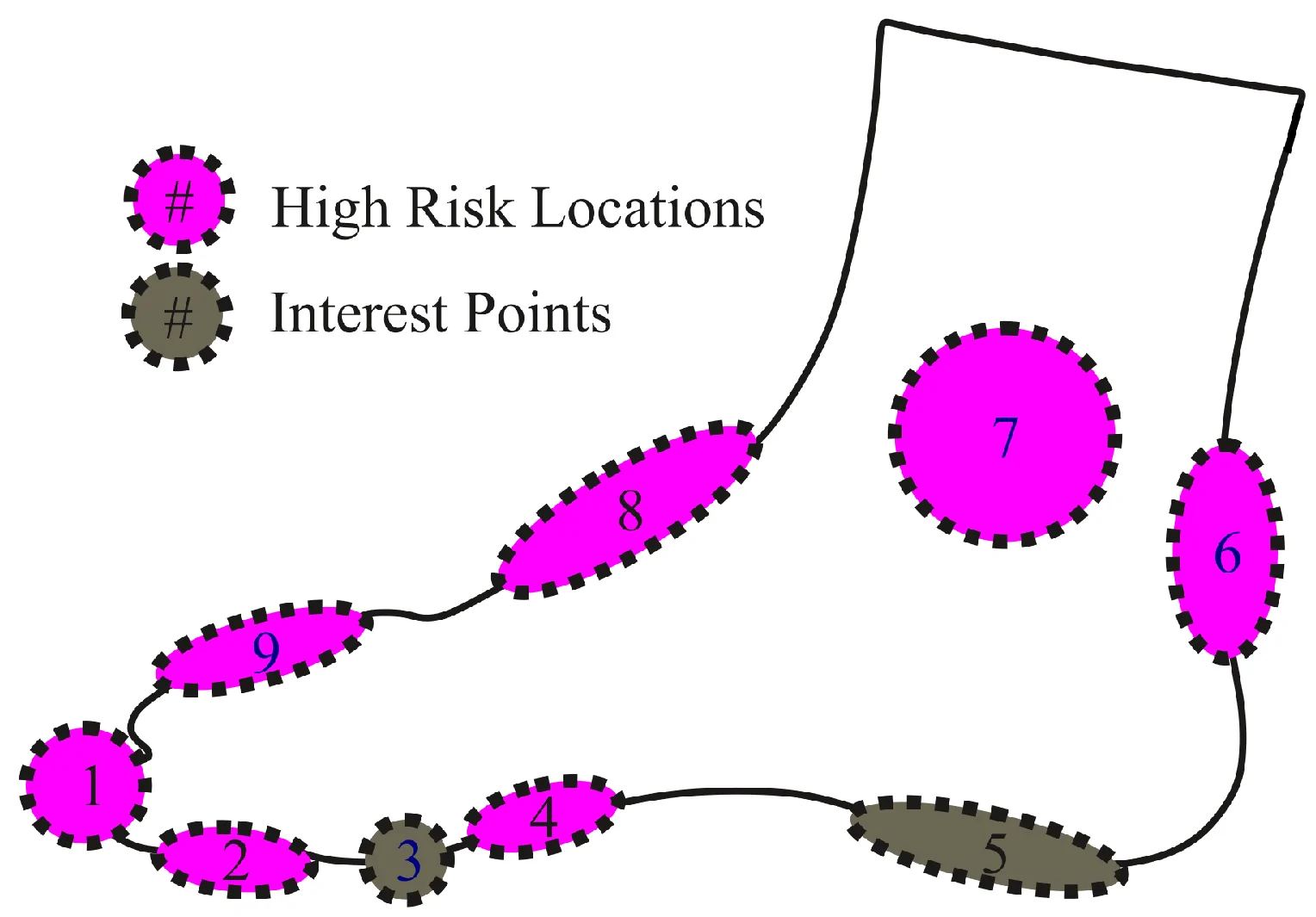
Locations on the foot at high risk of developing pressure ulcers. (1) Toe apices, (2) interphalangeal joints, (3) metatarsal head, (4) plantar metatarsal, (5) heel pad, (6) Achilles heel, and (7) malleolus medial, (8) foot dorsum and (9) toe dorsum. Grey interest points, pink other high risk locations.

During clinical engagement, podiatrists responded positively to the concept and highlighted a clear need for the technology. The workshop identified essential requirements for the final system, including biocompatibility due to the risk of fissures in feet; a low profile due to the risk of inducing skin damage that might lead to DFU formation due to increased applied pressure and the need for the system to be washable [42]. During engagement, clinicians identified the need for localised measurement of areas with a high risk of DFU formation on the foot (Figure 2), (1) toe apices (tips of the toe), (2) interphalangeal joints, which cover the metatarsal phalangeal joints, the proximal interphalangeal joints (PIP), and distal interphalangeal joints (DIP) (toe joints), (3) metatarsal head (toe base), (4) plantar metatarsal (bones behind toes), (5) heel pad, (6) Achilles heel, and (7) malleolus medial (ankle bony prominence). During the discussion, clinicians identified a potential interest in the technology, with particular interest in providing information on regions where tracking is not available through insoles, such as (8) foot dorsum (top of the foot) and (9) toe dorsum (top of the toes). Monitoring dorsal foot strain could help identify the degree to which the shoe laces have been tightened and whether action needs to be taken to change the tightness. The Achilles heel (6) and malleolus (7) of the high-risk regions cannot be tracked using current technologies. In this study, the metatarsal head (3) and heel pad (5) were selected as high risk locations. Future development would increase the array size to cover the dorsal surface.

In addition to the biocompatibility, localisation and a low profile, three key principles were selected to guide decision-making during system development: Technology readiness; to fast-track potential patient-impact solutions, we prioritised the implementation of commercially available technology. Production compatibility; to ensure the designed system could be applied to real-world settings, we ensured the final design was compatible with industrial textile production techniques. Scalability; in addition to compatibility with textile production, we set out to ensure the overall solution was scalable. By including this during our early-stage research, we hope it will aid in reducing the per-unit cost and, therefore, the associated “burden” of using STEPS.

### 2.2. The STEPS Concept

**Figure 3:**
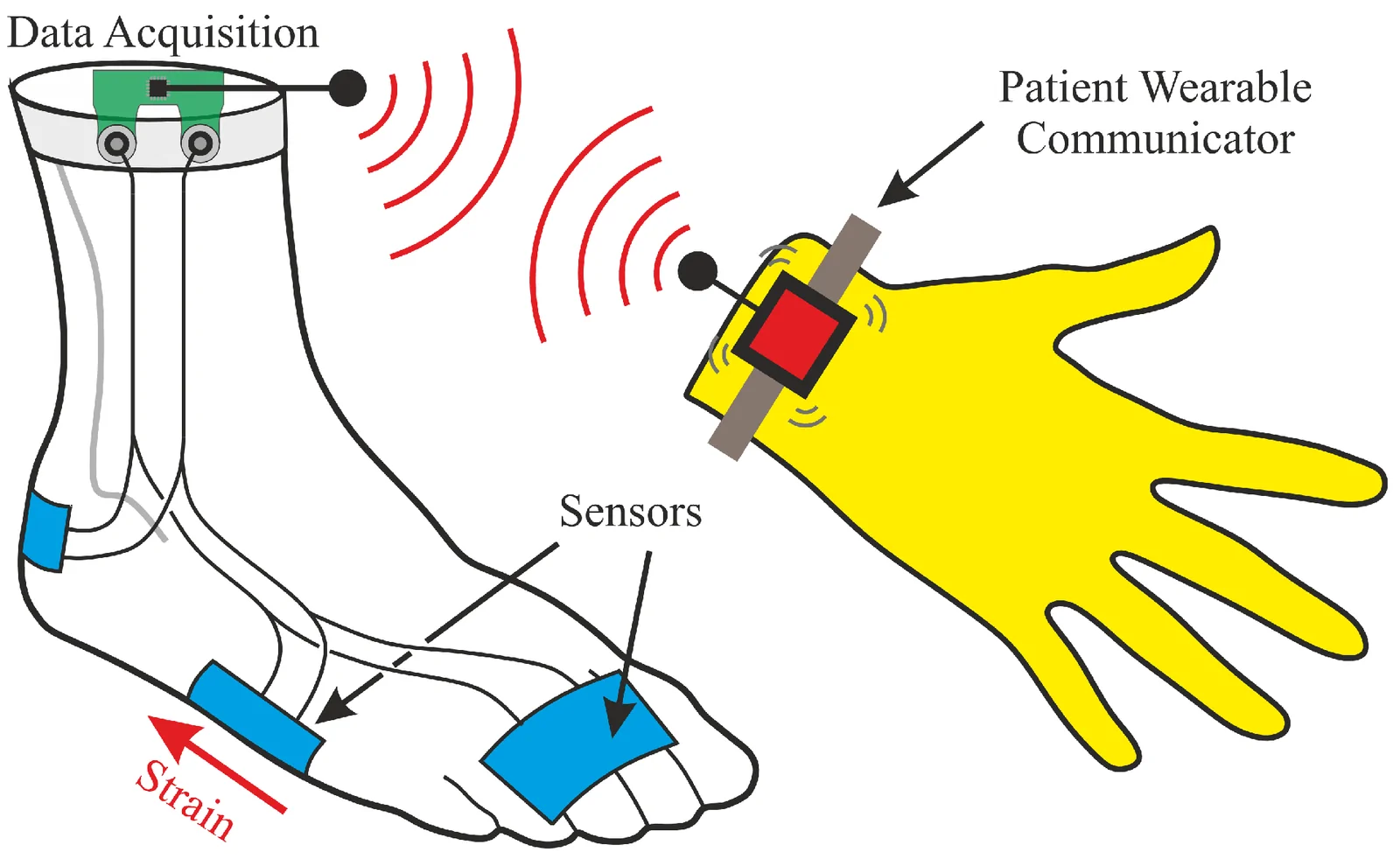
Shear Tracking for Enhanced Prevention Sock (STEPS) concept.

Pressure injuries are classically associated with sustained, unrelieved loading, with immobility a principal risk factor [25], whereas in the diabetic foot, mechanical tissue stress, comprising both plantar pressure and shear, accumulates over repetitive cycles of weight-bearing activity [49]. Having defined our design approach and user requirements, we set out to design a system capable of detecting shear that could seamlessly integrate within a mass-produced textile structure, in this case a sock. In line with the production-compatibility and scalability requirement set out above, sock production was undertaken on the Sangiacomo Star-D (Santoni SPA, Brescia, Italy), an industrial circular knitting machine representative of the equipment used for commercial-scale sock manufacture. The sensors detecting the applied shear would be read by a microprocessor-based data acquisition unit placed at the welt of the socks. In future development, the microprocessor could then be used to determine risk status and transmit this to a mobile communication device or app to notify the patient of the detected conditions (Figure 3).

At this stage, the research focuses on the data acquisition system and wearable development, with future work required to identify the risk status and investigate how this is communicated to the user. As highlighted in our design process, further PPIE will be necessary to identify the format this should take to gain the most benefit for the user. The following section will explore different aspects of the STEPS system in greater detail.

In our earlier work investigating sensor design, we explored various sensing methodologies that could be implemented to detect shear [50]. In line with the requirements set out above, the final design implemented infers shear detection through the proxy of textile (sock) strain. The textile stretches in response to a shear force applied to the sock’s surface. Due to the applied load coupled to the observed shear, it can be expected that the resulting movement would occur in both the textile and the underlying tissue. The assumption is that the strain measured due to shear will be similar for the textile and tissue. Therefore, textile properties are fundamental for STEPS performance.

### 2.3. Sock Textile Design and Evaluation

The sock substrate of STEPS must meet the needs of people with diabetes at risk of DFU formation. Although a range of products on the market advertise themselves as “diabetic socks”, at the time of writing they are not considered medical devices, although a medical benefit is implied by often including the term “diabetic” in the name. They are therefore exempt from regulation by bodies such as the EU MDR or the US FDA. The result is no clear definition of what constitutes a “diabetic sock”. A recent systematic review by Venkatraman et al. [51] analysed the current literature and commercially available products. Informed by PPIE work [42], their review and testing, Venkatraman et al. [51] set out technical requirements for a “diabetic sock”. The findings of these two studies were reviewed with clinical experts and used to develop the sock design used to produce STEPS.

Sock design improvements include a tapered profile to ensure a close but not tight fit, as people with diabetes identified that they often have swollen lower extremities, where a tight welt (top of the sock) further reduces blood flow and comfort and therefore adherence. The toes and heel cup use terry knit structures for additional comfort, and the welt was enlarged to distribute pressure over a greater area. The knit structure is otherwise homogeneous along the length of the sock, with no banded reinforcement zones.

**Figure 4:**
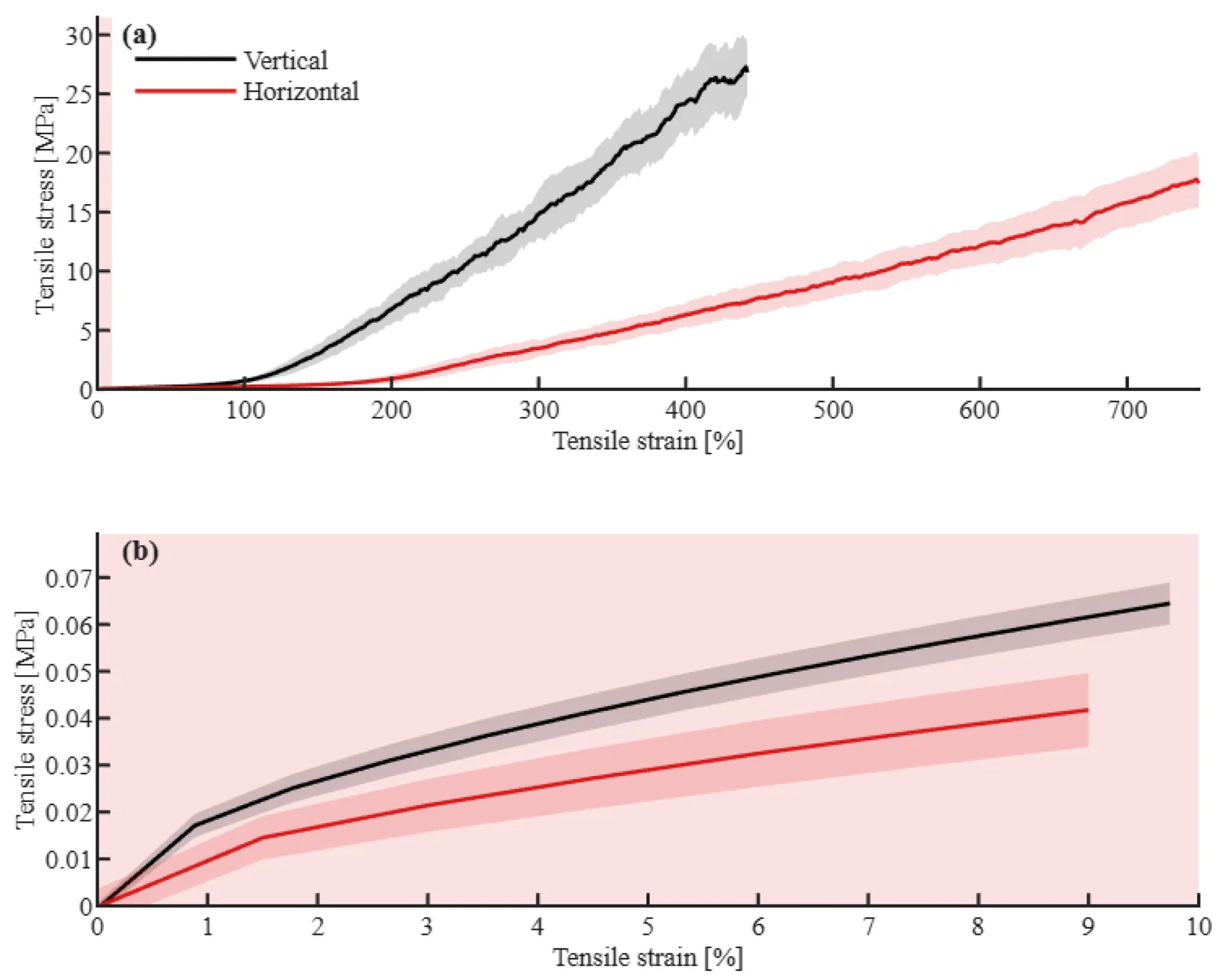
Sock mechanical performance for different orientations, shown as the mean of *n* = 15 specimens with ±1 standard deviation bands. (**a**) Full response to failure; (**b**) the expected sensor operating range (0–10% strain, shaded red), over which the vertical orientation is 1.43 × stiffer than the horizontal.

Because STEPS infers shear from textile strain, the mechanical response of that structure directly conditions sensor output, and any directional dependence in the knit would propagate into the measured signal. During production, the socks are heat-set on a form template and then washed once. Three sample socks were subjected to destructive mechanical stress–strain tests in a tensile testing machine (Instron 5943, High Wycombe, UK) to understand their strain properties in vertical (along the leg) and horizontal (circumferential) orientations. The Instron 5943 was equipped with 250 N pneumatic jaws (Instron 2712-052) and a 500 N load cell (Instron 2580-500N). Custom jaw faces were produced to secure the sock samples and prevent them slipping between the jaws. Five samples per sock per orientation were tested in tension to failure (*n* = 15 per orientation), giving the stress–strain response shown in Figure 4.

The socks were manufactured using commercial production equipment, resulting in a low inter-sample variation. Mean stiffness over the sensor operating range differed by 0.9% between the three socks in the vertical orientation (0.561, 0.560 and 0.565 MPa; one-way ANOVA *p* = 0.97) and by 11.2% in the horizontal orientation (0.388, 0.415 and 0.371 MPa; *p* = 0.56), with no statistical difference between socks in either case (MATLAB R2025a, The MathWorks Inc., Natick, MA, USA).

The two orientations, however, are not equivalent. Over the 0–10% strain range accessible to the sensor, the vertical orientation was stiffer than the horizontal (0.562 ± 0.034 MPa versus 0.392 ± 0.062 MPa, a ratio of 1.43; Welch’s *t*-test *p* < 0.001), with no overlap between the two distributions (minimum vertical specimen 0.506 MPa, maximum horizontal 0.451 MPa). Expressed as applied load, reaching 10% strain required 0.70 ± 0.05 N vertically compared to 0.47 ± 0.09 N horizontally, a 48% difference. The horizontal orientation exhibited greater variation with a coefficient of variation 15.8% compared to the verticals 6.1%. The knit structure is therefore homogeneous in construction but mechanically anisotropic in response.

This has a direct consequence for STEPS: an identical applied shear would produce a different textile strain, and hence a different sensor output, depending on the orientation of the sensor relative to the knit structure. Consequently silicone reinforcement [Ecoflex 30-00, Smooth-On Inc., Macungie, PA, USA] was applied at the sensing sites to impose a defined and repeatable local stiffness region, designed to decouple sensor response from knit direction and from the specimen-to-specimen variation observed in the more compliant orientation. Additionally the silicone is intended to provide protection to the sensor during a wash cycle.

It should be noted that although the socks were stretched to failure, the fabric structure and elasticity lent by the Lycra yarns mean the total extension achieved is far greater than the sensor will experience in use. Failure occurred at 496 ± 32% vertically and 1100 ± 118% horizontally, some 50 and 110 times the sensor’s 10% active range, respectively, so the textile is never the strain-limiting element of the system.

### 2.4. Sensor Design and Manufacture

**Figure 5:**
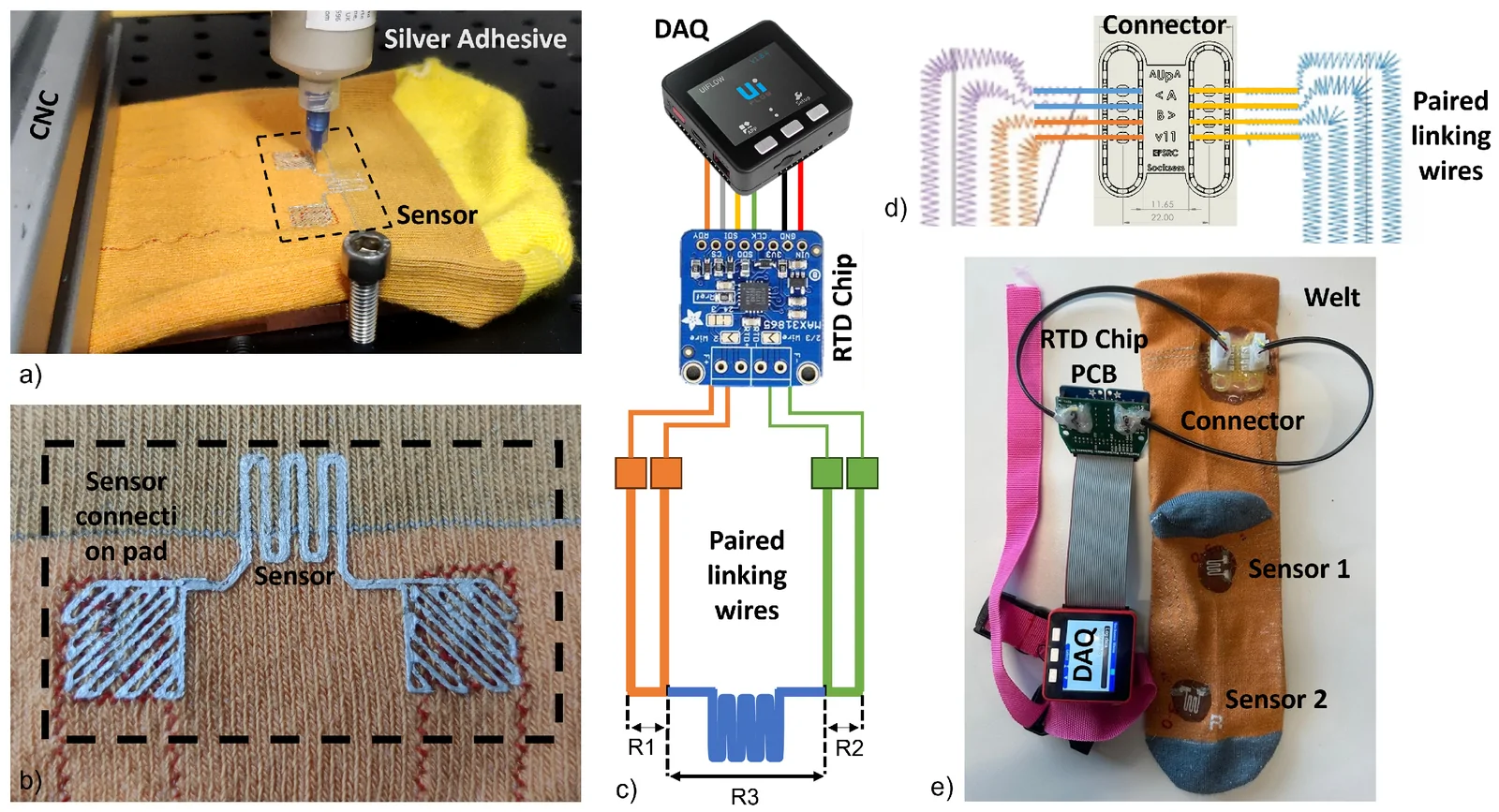
Prototype of the STEPS smart sock. Highlighting (**a**) the sensor production process, (**b**) printed silver sensor, (**c**) schematic diagram of the sock connectivity, (**d**) welt connector and sensor paired link wires connector integration, and (**e**) STEPS prototype showing two strain sensors and connected electronics.

The sensor design adopts a typical strain gauge format, with six turns and traces of 10 mm. The sensor was 3D-printed with conductive silver adhesive (Dycotec DM-SAS-10010, Dycotec Materials Ltd., Calne, UK) and manufactured using a CNC (WorkBee Z1+ CNC, Ooznest, Brentwood, UK), connected to a precision syringe dispenser (Ultimus V High Precision Dispenser, Nordson, Westlake, OH, USA) (Figure 5a). Details on the development and characterisation of these sensing elements can be seen in previous work by the authors [50]. This paper focuses on integration of the sensing element into a prototype wearable device, together with evaluation in a proof-of-concept study (Figure 5b).

### 2.5. Electronics and Data Acquisition

Sensor readings were captured through repurposed platinum resistance temperature detectors (MAX31865, Adafruit, New York, NY, USA) that provide resistance measurement at a quantisation resolution of 0.013 Ω for the chip’s measurable range of 0–430 Ω.

To bridge the gap between the sensor and the data acquisition unit at the socks welt, several methods were investigated, including: silver ink traces, embroidered conductive yarns, and ceramic-coated copper. Ceramic-coated copper wires were used during early prototyping and for the proof-of-concept. Although this does not meet our requirements for removing ridges from the device, we have focused on healthy participant testing at this stage. The RTD chip enables paired connectivity so that the resistance of the linking wire can be measured and accounted for in resistance readings (Figure 5c) R1 and R2, accounting for the conductivity changes observed between the copper wire and the conductive yarns.

The MAX31865 was operated in single-conversion mode with a 50 Hz noise filter. In the two-sensor configuration used in the case study (Section 3), resistance data was collected at 6 Hz. The IMU was sampled at 40 Hz and all data were recorded synchronously at this rate. It is noted that this resistance sampling rate is lower than the 50–100 Hz typical of commercial plantar-pressure systems and the minimum 50 Hz recommended for capturing loading dynamics during walking [52,53]. Consequently, the current sampling rate is insufficient to resolve high-frequency transient loading events during heel-strike and toe-off. However, the system provides approximately 10–12 resistance measurements per gait cycle (approximately 1.75 s), allowing evaluation of baseline drift, repeatability and peak-to-peak load response. The IMU data are used only for the segmentation of gait events, for which accelerometer rates as low as 31.25 Hz are sufficient [54]. As this study aims to demonstrate the feasibility of the technology rather than a clinically validated measurement system, further development is required before clinical implementation. Initial firmware optimisation using continuous MAX31865 conversion, asynchronous acquisition and buffered data writes, following Wang et al. [55], could increase the resistance sampling rate to approximately 50 Hz, although this would only marginally meet the recommended minimum. A subsequent hardware redesign, replacing the breakout boards with a single custom PCB, would reduce the system’s form factor and provide an opportunity to upgrade the data-acquisition hardware. In particular, transitioning to an ADC supporting ratiometric four-wire resistance measurement, such as the ADS1220 [Texas Instruments, Dallas, TX, USA], which offers data rates of up to 2000 samples/s, could substantially increase the sampling rate while retaining the four-wire measurement approach and compensation for lead resistance.

Sensor resistance was not converted to a physical strain value in this study. Bench characterisation of the sensing element [50] indicated that the resistance-strain response is sensor-specific rather than following a single generalisable relationship. A per-sensor calibration is further complicated by the resistance drift reported in [50], where it was attributed to micro-fractures. As the sensor’s resistance-strain relationship shifts as it drifts, a calibration curve established prior to use cannot be assumed to remain valid over the sensor’s service life.

The DAQ unit is connected to a microcontroller (Core3, M5Stack Technology Co. Ltd., Shenzhen, China), which provides data logging and a user interface (Figure 5c). Firmware was developed in the Arduino IDE (version 2.3.2; Arduino S.r.l., Monza, Italy). The microcontroller has an inertial measurement unit (IMU) which was used to collect acceleration data. Synced with sensor data collection, the acceleration data supports the identification of gait cycles and the interpretation of the sensor response. In this proof-of-concept development stage, breakout boards were implemented. A custom PCB provided a compact connection between the DAQ unit and the microcontroller. Future work will investigate miniaturisation to reduce the unit’s dimensions, weight, and inertial properties to improve user comfort.

In electronics, a common challenge is the transition from flexible to rigid structures. In this instance, from the wire termination point at the welt to the data acquisition unit. Currently, there are limited connectors available for textile-electronics interfaces, therefore the authors designed their own. A few factors are ensuring user comfort and minimal rubbing or sharp edges, as this could damage the user’s skin. Therefore, the authors opted to avoid adhesives that would cause the textile to stiffen. A custom PCB was designed that aligned with the termination points of the linking conductors (Figure 5d). Small quantities of the same Dycotec adhesive (DM-SAS-10010, Dycotec Materials Ltd., Calne, UK) used for the sensors was used to provide a flexible coupling between PCB and ceramic-coated copper. The same methods can be used for embroidered traces. Over such short distances, the silver adhesive provides excellent connectivity with minimal resistance. Silicone (Ecoflex 30-00, Smooth-On Inc., Macungie, PA, USA) was poured over the connector to provide mechanical reinforcement (Figure 5e), a matrix of through holes on the PCB aided silicone ingress to ensure a secure connection. Placement (Figure 5) of Sensors 1 and 2 aligned with the heel pad and metatarsal head, respectively (Figure 2 location 5 and 3 respectively).

Based on PPIE feedback, the ability to wash the system was identified as a key factor in long-term usability. STEPS was designed for disassembly to facilitate the removal of components incompatible with the typical wash cycle, in this case, all components above the welt connector (Figure 5e).

## 3. Case Study

A single-participant proof-of-concept case study of wearing the STEPS sock was conducted to evaluate the ability of STEPS to measure strain during gait.

### 3.1. Methods

The case study undertaken for this article received approval from the Ethics Review Committee of the Faculty of Engineering and Physical Sciences of the University of Leeds under EPS FREC 2025-2201-3414. Eligibility criteria included being >18 years old, capable of walking without assistance and having not been diagnosed with diabetes or any other foot health condition. Participants completed a screening questionnaire prior to acceptance into the study. As a proof-of-concept study, a single participant was recruited, common practice in the development of wearable systems. The participant was a 188 cm male in their 30s with a BMI of 29.7, within the overweight range typical of people with type 2 diabetes [56], size 12 feet and no underlying medical conditions. The participant received a custom STEPS prototype sock and a corresponding non-sensing sock, both properly sized to fit their foot. The participant completed a series of ten gait cycles on a flat walkway at a self-selected pace, starting with the STEPS foot, repeated over ten repetitions.

The STEPS resistance sensor data was reconstructed from the ∼6 Hz sensor updates by shape-preserving interpolation (pchip), with zero-value dropouts (0.87% of Sensor 1 samples) excluded. Noise was estimated as the standard deviation of the detrended resistance during standing periods between repeats. A ten-second standing period was included between repeats to establish an inactive baseline for drift correction.

Triaxial acceleration from the microcontroller’s onboard IMU was collected at 40 Hz to aid in gait cycle segmentation from repetitions. It was filtered by a low-pass 4th-order Butterworth at 5 Hz. The magnitude of acceleration data was analysed to identify gait markers (e.g., heel-strike) and segment each repetition into individual gait cycles; the first and last cycles of each repetition were discarded, leaving eight steady-state cycles per repetition (80 cycles per sensor in total). In each repeat, a standing baseline was subtracted to correct for sensor drift before cycles were time-normalised to 100 points to allow direct comparison.

### 3.2. Results

**Figure 6:**
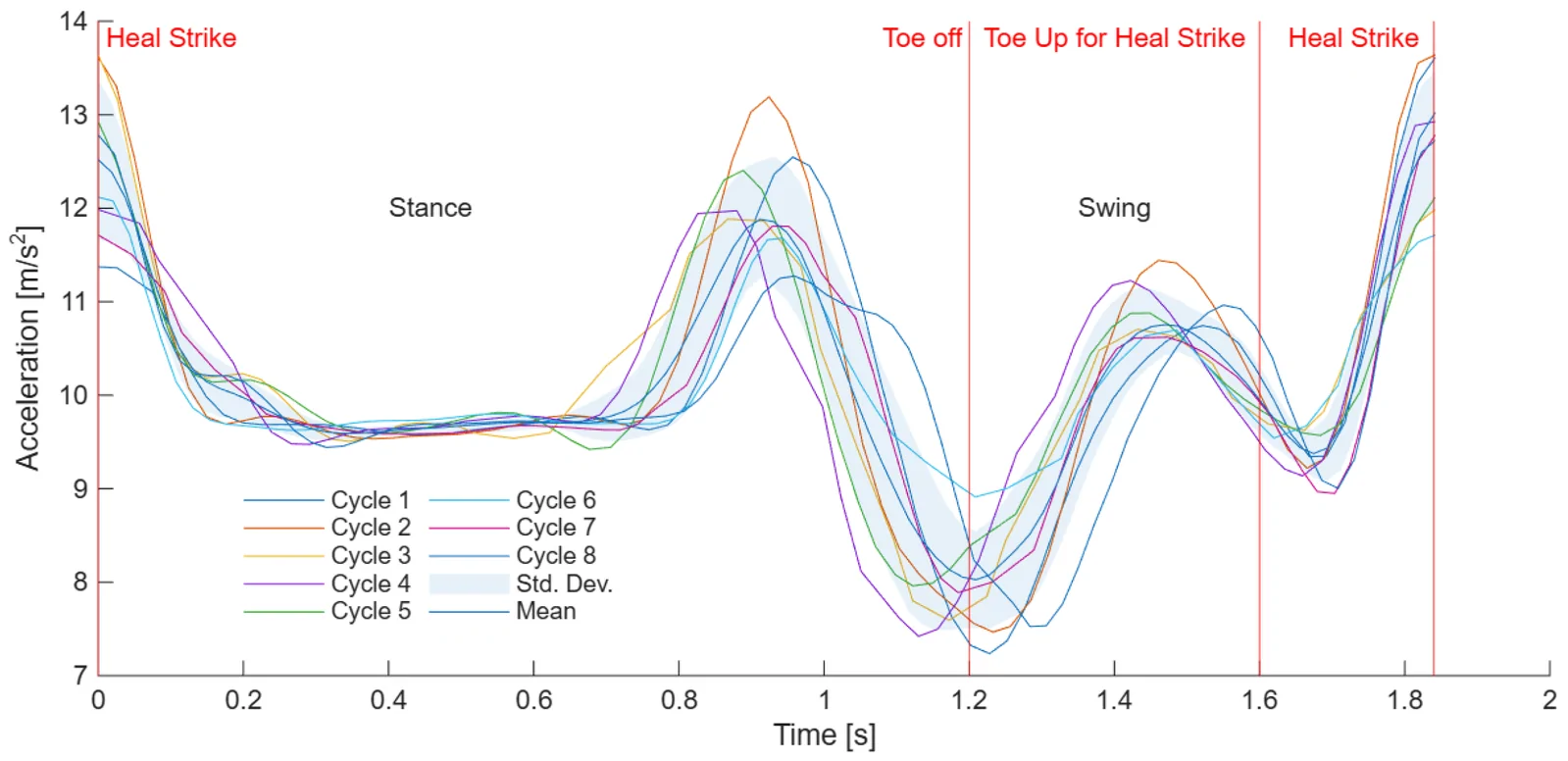
Accelerometer data from data acquisition unit across a single repeat showing the 8 gait cycles (Cycle 2–9) with a mean and standard deviation.

The acceleration magnitude showed strong repeat-to-repeat consistency (between-repeat SD = 6.6% of range, mean correlation *R* = 0.91, moving-window correlation *R* = 0.71), supporting its use as a stable reference for gait-cycle segmentation throughout this analysis (Figure 6).

**Figure 7:**
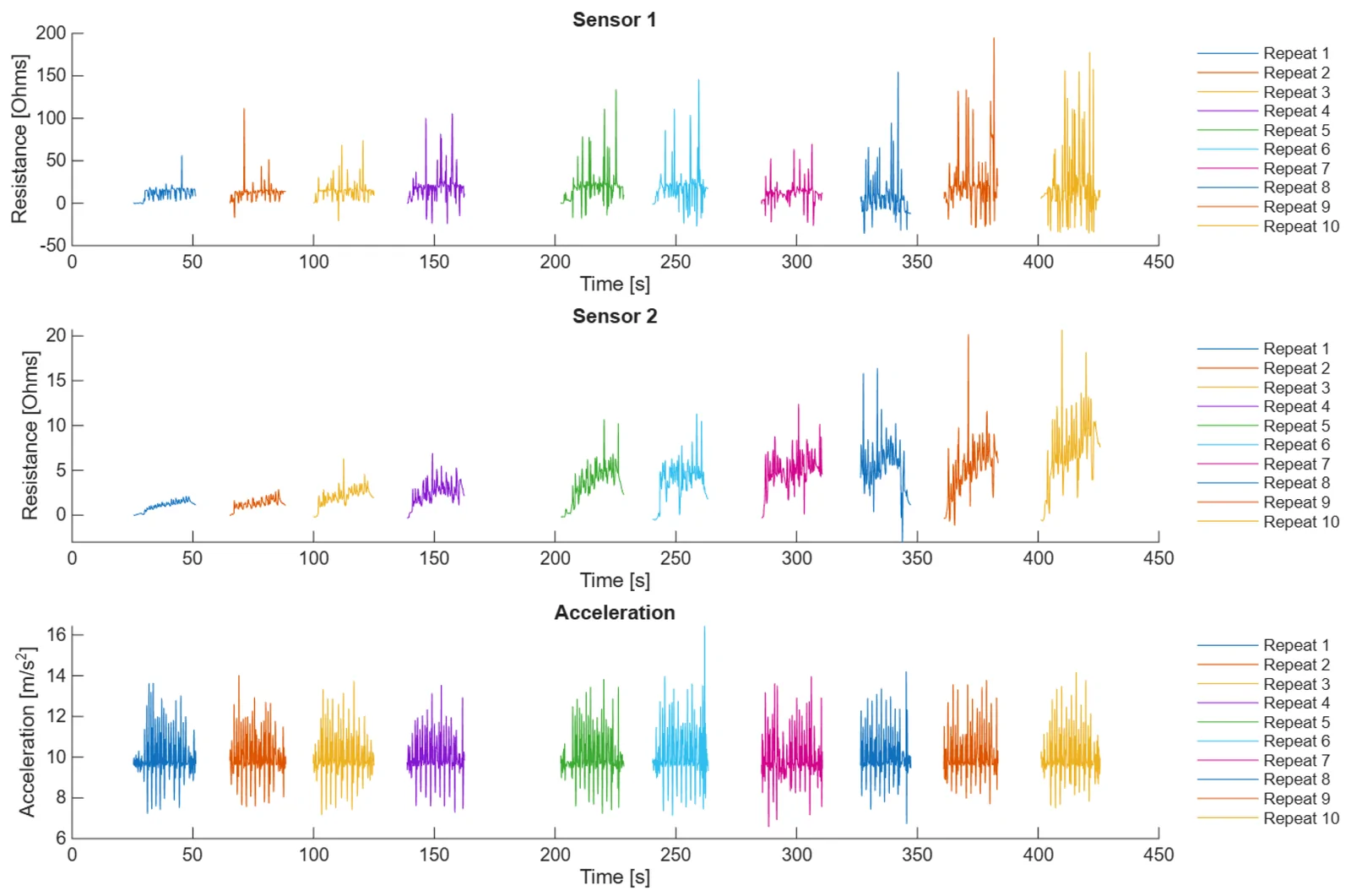
Resistance response for Sensor 1, Sensor 2 and acceleration magnitude across the ten repeats, illustrating baseline drift between repeats. Gait events identified from the acceleration data are shown against the resistance response for a single repeat in Figure 8.

Sensor 1 produced a larger resistance response per gait cycle (63.5 ± 45.1 Ω) than Sensor 2 (4.42 ± 3.76 Ω). Sensors 1 and 2 functioned over a signal range of 224.3 Ω and 27.2 Ω respectively (Figure 7). The noise floor, measured while stationary, was 3.53 Ω for Sensor 1 and 0.29 Ω for Sensor 2, against overall mean cycle amplitude of 224.3 Ω and 27.2 Ω, respectively. When normalised by range, this equates to 1.6% and 1.1% respectively. The high standard deviation relative to the mean for both sensors reflects the natural cycle-to-cycle variability inherent to overground walking rather than a fixed, repeatable amplitude. Sensor 1 also showed higher noise relative to its range (1.6% versus 1.1%) and isolated dropouts, suggesting a difference in sensor or interface condition between channels that cannot be resolved with two sensors on a single participant (Table 1).

Consistent with the dynamic drift previously reported for the sensor during bench-top cyclic loading [50], both sensors exhibited an upward drift in baseline resistance over the course of ten repeats (Figure 7). Sensor 1 drifted by 1.59 Ω/repeat (61.9% of its initial baseline, $R^{2}$ = 0.55), while Sensor 2 drifted by 0.74 Ω/repeat (153.1% of its initial baseline, $R^{2}$ = 0.98). Although the proportional drift for Sensor 2 is larger, its smaller absolute baseline means this remains a modest 6.4 Ω change, spanning 2.5% of the DAQ’s 0–430 Ω measurable range, compared with 8.2% for Sensor 1.

By extrapolating the linear drift trend, Sensor 1 and Sensor 2 would not be expected to saturate the data acquisition chip for further 2470 and 5630 gait cycles, respectively. This is comparable to the 2275 (±562)-cycle service life estimated during the cyclic loading on the bench for Sensor 1 and exceeds it for Sensor 2 [50]. Given the poorer linear fit for Sensor 1 ($R^{2}$ = 0.55), its estimated service life should be treated as indicative rather than as precise.

Sensitivity is determined for each sensor as the peak-to-peak resistance change within each gait cycle *i* (Equation (1)).

(1) $$S_{i}=\max_{t}R_{i}\left(t\right)-\min_{t}R_{i}\left(t\right),$$

reported in Table 1 as the mean $\bar{S}$ and standard deviation $\sigma_{S}$ in all *N* = 80 gait cycles (8 cycles × 10 repetitions).

For Sensor 1, the noise floor 3.53 Ω is 5.6% of the mean per-cycle response 63.5 Ω; for Sensor 2, the noise floor 0.29 Ω is 6.6% of the mean per-cycle response 4.42 Ω. In both cases the noise floor is an order of magnitude smaller than the typical per-cycle strain response, indicating that individual strain events are resolvable above the noise floor for both sensors.

**Figure 8:**
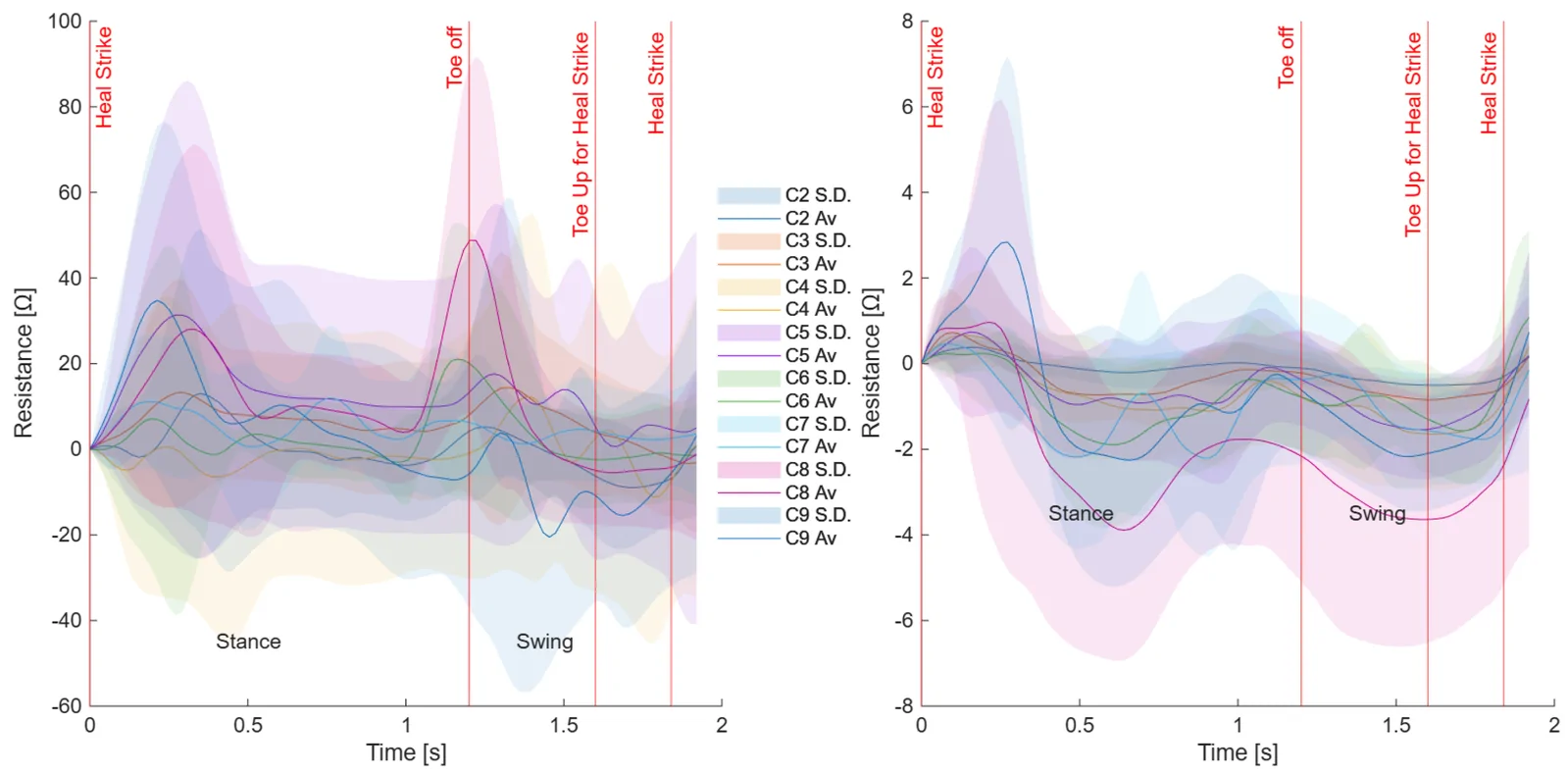
Mean (±SD) gait-cycle-normalised resistance response for each sensor across the ten repeats, illustrating within- and between-repeat variability.

Cycle-to-cycle (within-repeat) variability exceeded repeat-to-repeat (between-repeat) variability for both sensors, corresponding to 71.8% vs. 24.9% of Sensor 1’s mean cycle amplitude, and 43.5% vs. 39.1% of Sensor 2’s dynamic range (Figure 8). Indicating that both sensors reproduce a consistent response throughout the ten repetitions performed, Sensor 2 showing the stronger repeat-to-repeat consistency (mean pairwise correlation *R* = 0.78, moving-window correlation *R* = 0.65) compared to Sensor 1 (*R* = 0.41 and *R* = 0.30, respectively). The moving-window correlation, which assesses repeatability at each point of the normalised gait cycle rather than as a single whole-cycle value, followed the same pattern, suggesting Sensor 1’s reduced repeatability is distributed across the gait cycle rather than localised to a specific phase (e.g., heel-strike or toe-off).

Comparing the two sensor channels directly, resistance responses showed negligible correlation (*R* = 0.07, RMSE = 26.7 Ω). This is expected, given the two channels correspond to independent sensor sites on the sock and demonstrates that each channel captures a distinct, location-specific loading pattern during gait. In the absence of an independent calibrated reference standard as set out in Section 2.5 (e.g., a load cell), this comparison reflects inter-sensor consistency rather than absolute measurement accuracy; establishing traceable accuracy against a reference remains a priority for future calibration work.

## 4. Discussion

This proof-of-concept case study demonstrates that the STEPS prototype can measure repeatable gait-induced strain (via resistance change) through two independently sited resistive sensors integrated within a sock. For both channels, the noise floor was small relative to the typical per-cycle strain response (5.6% and 6.6% of the mean cycle amplitude for Sensor 1 and Sensor 2, respectively), and both showed repeatability within the gait-cycle. It is noted that there has been a reduction in repeatability in the transition from bench top testing to wearable integration [50]. This reduction in repeatability, particularly for Sensor 1 (mean between-repeat correlation *R* = 0.41, compared with *R* = 0.78 for Sensor 2), likely reflects the additional variability inherent to overground gait relative to a single-axis bench loading regime, including natural intra-stride variation, donning effects, and the coupling of shear with normal load and skin-textile friction that cannot be isolated on the bench. Repeatability was reduced relative to bench testing, as anticipated under the multiaxial and less controlled loading conditions of the in-shoe environment. In particular, Sensor 1 showed low repeatability (R=0.41); therefore, these results demonstrate proof-of-concept operation but do not establish measurement repeatability.

In the literature, the magnitudes of plantar shear stress during gait range from approximately 18 to 158 kPa depending on the measurement site and population [23], with higher values associated with a history of DFU (135.3 ± 60.6 kPa) compared to diabetic neuropathy alone (86.4 ± 30.3 kPa) [57]. Comparable in-shoe wearable shear sensors have reported peak shear stresses of 66.5–152.6 kPa (anteroposterior) and 28.4–128 kPa (mediolateral) during walking [58]. A direct quantitative comparison with the resistance changes reported here is not yet possible, as resistance has not been converted to a calibrated strain or stress value. It is not possible to infer clinical thresholding until calibration has been applied and validated.

Reflecting on the three guiding principles (readiness, production compatibility and scalability) set out in Section 2, the case study supports technology readiness: built from commercially available components, the system successfully collected strain data outside the bench setting used in prior characterisation [50]. Production compatibility and scalability are not directly assessed by this single-participant proof-of-concept. The influence of textile performance on sensor output (Section 2) requires characterisation to support future calibration work ahead of longer-duration wear trials.

It should be emphasised that the strain, measured here, is not itself the direct cause of the formation of DFU; rather, it is used as a proxy for the shear component of the coupled normal-shear loading regime implicated in ulcer formation [22,23]. The results presented demonstrate that STEPS can resolve gait-induced strain events, supporting its use as a shear proxy, but do not establish a link to the normal (pressure) component also relevant to the risk of DFU; combined shear and pressure sensing remains a target for future development.

Several issues specific to the application in diabetic foot assessment warrant discussion in light of the results presented. Both sensors exhibit sustained upward drift, 1.59 and 0.74 Ω per repeat for Sensor 1 and Sensor 2, consuming 8.2% and 2.5% of the DAQ’s usable range within just ten repeats.

Drift is an established property of this sensor and has been attributed to micro-fracture accumulation under controlled cyclic loading [50]. The near-linear drift of Sensor 2 ($R^{2}$ = 0.98) is consistent with steady accumulation of this kind rather than mechanical settling of the sock, which would be expected to diminish over the session. Silicone reinforcement constrains sensor movement relative to the textile and, together with plantar pressure, is expected to limit migration relative to the skin [50]. Per gait cycle, drift was 0.037% and 0.017% of the measurable range for Sensor 1 and Sensor 2, respectively. Sensor 1 agrees with the 0.039–0.045% observed during bench testing, whereas Sensor 2 exhibited lower drift [50]. Responses also differed from the bench-defined 0–20 Ω range of interest: Sensor 2 approached this range (27.2 Ω), while Sensor 1 exceeded it by an order of magnitude (224.3 Ω). This difference may reflect the multiaxial and less controlled stresses imposed during use compared with the single-axis bench-loading regime. Sensor 1 also exhibited less linear drift ($R^{2}$ = 0.55) and greater within-repeat variability relative to mean cycle amplitude (71.8% versus 43.5%), indicating that the disparity was not solely caused by its larger absolute response. Site-specific effects are also plausible because the two locations experience different strain magnitudes and directions. Given the knit anisotropy described in Section 2, sensor orientation and local silicone reinforcement may further affect strain transmission. These contributions cannot be separated in this two-sensor, single-participant study and require repeated placements across participants and sensing sites.

Connector or wire integrity is an unlikely explanation because connection loss causes measurement-chip saturation, which was not observed, and both channels remained within the 430 Ω measurement limit. Isolated zero-value dropouts from Sensor 1 accounted for 0.87% of samples. Excluding them changed the noise estimate by only 1.5% and did not affect the signal range, indicating that they do not explain the observed channel differences.

Additional material development is required for reliable long-term deployment and prior to calibration work, particularly given that people with diabetes may wear STEPS for considerably longer and more variable periods than those tested here. Drift in this sensor has been attributed to micro-fracture accumulation [50], and this type of physical damage ultimately limits the sensor lifetime, as algorithmic correction cannot recover irreversible degradation such as fracture or delamination [59]. Given the low drift linearity observed for Sensor 1 ($R^{2}$ = 0.55), a simple linear correction would not be sufficient and a non-linear calibration approach may be required.

Algorithmic approaches can address the recoverable effects of non-linearity, hysteresis, rate dependence, and progressive drift. Sequence models that use the resistance time history, rather than a single instantaneous value, have been shown to be effective. Oldfrey et al. applied a long short-term memory network to commercial stretch sensors, reducing the mean absolute prediction error from 19–31% using linear regression to 11–19% [60]. Li et al. developed a physics-guided transformer calibration framework for their soft strain sensor that compensated for non-linearity, hysteresis and cycling attenuation over 20,000 cycles, using the cycle count as an input along with the resistance history [59]. For STEPS, the gait cycle segmentation from the IMU already provides this usage history. Similar time-history approaches have also been applied to the calibration of pressure-sensing insoles [61]. Applying these methods to STEPS would require a time-resolved strain measurement for training, but this is lacking in the current system configuration. As drift alters the resistance-strain relationship over time, calibration could incorporate usage history [59], with the STEPS IMU identifying individual gait cycles to provide the cycle count.

Following materials development, each sensor would first be characterised on the reinforced sock textile in its installed orientation, under cyclic loading within the 0–10% strain range at gait-representative rates and including a donning pre-strain, to establish its resistance–strain relationship, hysteresis and rate dependence. Testing reinforced sites in both knit orientations would verify that the silicone reinforcement decouples sensor response from the anisotropy of the textile, with the orientation-specific textile stiffness applied where conversion to stress is required. Calibrated outputs would then be validated against a reference plantar shear measurement, using an in-shoe shear sensing system [55] or a bench rig applying known shear loads, before longer-duration and repeated-donning trials assess whether calibration remains valid over time and between fittings.

Differences in foot shape: the negligible correlation observed between the two sensor channels (*R* = 0.07) indicates that each captures an independent, site-specific loading signature during gait. While desirable for the localisation goals of STEPS, this also implies that sensor response cannot be assumed to generalise across sock locations, or, by extension, across the atypical foot shapes and deformities (e.g., claw foot) common in the target patient group [15]. This suggests that a baseline specific to each individual is likely necessary, rather than using a single global calibration applied across users.

Several areas of future work have been identified from this proof-of-concept evaluation. Foremost, the single-participant test reported here should be extended to the full multi-participant case study. Ongoing work investigating the optimisation of textile-based connectivity through embroidery or inline knitting by an industrial knitting machine will need to be implemented in future work before deployment with any at-risk populations.

Given the observed micro-fracture-linked drift behaviour [50], establishing a robust calibration approach, potentially incorporating drift compensation or periodic recalibration, remains a priority for future work. Detailed investigation of the impact of washing is required with a possible impact on textile and silicone quality with repeated cycles and related implications on sensor function and calibration. In parallel, material development is required to address the micro-fractures, increasing the sensor lifespan. Establishing measurement accuracy against an independent calibrated reference standard (e.g., a load cell) is also required, since the present validation could only assess consistency between the two sensor channels rather than absolute accuracy. Longer-duration wear studies, beyond the ten repetitions reported here, are needed to validate the projected sensor lifespan under continuous daily use and to characterise drift and saturation behaviour over realistic wear periods.

Data acquisition miniaturisation will reduce wearable weight, and inertial impact on user comfort, alongside further development of textile-based connectivity to replace the ceramic-coated copper wiring used in this proof of concept, and evaluation of the durability of silver-ink-based traces through repeated washing cycles. The terry-loop sections incorporated into the sock design will be evaluated for their potential to mechanically buffer sensor regions without compromising strain transmission. Further PPIE work is required to define the format of patient-facing feedback and risk communication.

The use of a single healthy participant limits the generalisability of these findings to people with diabetes, but this was not the purpose of the present study, which was intended as a proof-of-concept demonstration of the STEPS prototype. We acknowledge that this will not faithfully represent the future intended target population and this will be addressed in future work. More substantial differences may arise from peripheral neuropathy and its associated effects, including altered gait, increased plantar tissue stiffness, callus formation, and foot deformity, all of which may affect the magnitude and distribution of plantar strain. The noise floor, measured during stationary standing, reflects the sensor and associated electronics and is not expected to depend substantially on the wearer. However, noise relative to the per-cycle response will vary with strain amplitude at each measurement site and may therefore change under altered loading conditions. Similarly, the per-cycle drift rate of Sensor 1 was consistent with bench testing, suggesting that drift is primarily determined by the loading history of the sensor. Differences in load magnitude and cadence may therefore alter the rate of drift accumulation. Evaluation in the target population is required before the clinical utility of STEPS can be established.

## 5. Conclusions

This paper presents the development and proof-of-concept evaluation of STEPS, a strain-sensing sock for the prevention of diabetic foot ulcers. Building on the sensor characterised in previous bench work [50], the sensor was successfully integrated into the wearable device comprising a bespoke knitted sock, flexible connectivity, and a data acquisition system. A single-participant proof-of-concept test demonstrated that the resulting prototype can capture repeatable gait-synchronised strain signals from two independently located sensor channels at the bottom of the foot, with a noise floor below 10% of the typical strain response per cycle for both sensors. Repeatability differed between channels: Sensor 2 achieved a correlation between repeats of *R* = 0.78, whereas Sensor 1 reached only *R* = 0.41, indicating that consistent cycle-to-cycle response cannot yet be assumed between sensing sites. Baseline drift was present, but remained modest relative to the data acquisition system’s measurable range, spanning 2.5–8.2% of the available range over ten repeats, giving an estimated 2470–5630 gait cycles before the sensor reaches saturation and would need to be replaced. The two sensor channels showed negligible correlation with one another, confirming that each captures an independent, location-specific loading signature, a key requirement for the localisation goals of STEPS. Together, these results represent a promising first step toward a wearable system capable of continuous out-of-shoe plantar shear monitoring, meeting the technology-readiness principle set for this work while highlighting the calibration, wear, and validation work still required ahead of clinical deployment.

## Figures and Tables

**Table 1:** Summary of sensor validation metrics from the single-participant proof-of-concept gait test (10 repeats, 10 gait cycles per repeat with 8 cycles assessed).

Metric	Sensor 1	Sensor 2
Signal range (Ω)	224.3	27.2
Noise floor, stationary (Ω)	3.53	0.29
Drift rate (Ω/repeat)	1.59	0.74
Total drift (% of initial baseline)	61.9	153.1
Drift trend fit ($R^{2}$)	0.55	0.98
Est. gait cycles within DAQ measurable range (0–430 Ω)	2470	5630
Within-repeat variability (% of mean cycle amplitude)	71.8	43.5
Between-repeat variability (% of mean cycle amplitude)	24.9	39.1
Between-repeat correlation (mean *R*)	0.41	0.78
Moving-window correlation (mean *R*)	0.30	0.65
Sensitivity (Ω/gait cycle) [$\bar{S}\pm \sigma_{S}$]	63.5 ± 45.1	4.42 ± 3.76
Noise (% of mean cycle amplitude)	5.6	6.6
Drift rate (% of range per gait cycle)	0.037	0.017

## Data Availability

The original data presented in the study are openly available in Research Data Leeds Repository 10.5518/1649.

## Abbreviations

The following abbreviations are used in this manuscript:

DAQ	Data Acquisition
DFU	Diabetic Foot Ulcer
DIC	Digital Image Correlation
DIP	Distal Interphalangeal Joint
FDA	Food and Drug Administration
IMU	Inertial Measurement Unit
MDR	Medical Device Regulation
NHS	National Health Service
PCB	Printed Circuit Board
PIP	Proximal Interphalangeal Joint
PPIE	Patient and Public Involvement and Engagement
RMSE	Root Mean Square Error
SD	Standard Deviation
STEPS	Shear Tracking for Enhanced Prevention Sock
